# Myocardial Strain Measured by Cardiac Magnetic Resonance Predicts Cardiovascular Morbidity and Death

**DOI:** 10.1016/j.jacc.2024.05.050

**Published:** 2024-08-13

**Authors:** Sucharitha Chadalavada, Kenneth Fung, Elisa Rauseo, Aaron M. Lee, Mohammed Y. Khanji, Alborz Amir-Khalili, Jose Paiva, Hafiz Naderi, Shantanu Banik, Mihaela Chirvasa, Magnus T. Jensen, Nay Aung, Steffen E. Petersen

**Affiliations:** a William Harvey Research Institute, NIHR Barts Biomedical Research Centre, Queen Mary University of London, London, United Kingdom; b Barts Heart Centre, St Bartholomew’s Hospital, Barts Health NHS Trust, London, United Kingdom; c Circle Cardiovascular Imaging Inc, Calgary, Alberta, Canada; d Steno Diabetes Center Copenhagen, Copenhagen, Denmark; e Health Data Research UK, London, United Kingdom; f Alan Turing Institute, The British Library, John Dodson House, London, United Kingdom

**Keywords:** cardiac magnetic resonance, circumferential strain, longitudinal strain, radial strain, survival analysis

## Abstract

**Background:**

Myocardial strain using cardiac magnetic resonance (CMR) is a sensitive marker for predicting adverse outcomes in many cardiac disease states, but the prognostic value in the general population has not been studied conclusively.

**Objectives:**

The goal of this study was to assess the independent prognostic value of CMR feature tracking (FT)—derived LV global longitudinal (GLS), circumferential (GCS), and radial strain (GRS) metrics in predicting adverse outcomes (heart failure, myocardial infarction, stroke, and death).

**Methods:**

Participants from the UK Biobank population imaging study were included. Univariable and multivariable Cox models were used for each outcome and each strain marker (GLS, GCS, GRS) separately. The multivariable models were tested with adjustment for prognostically important clinical features and conventional global LV imaging markers relevant for each outcome.

**Results:**

Overall, 45,700 participants were included in the study (average age 65 ± 8 years), with a median follow-up period of 3 years. All univariable and multivariable models demonstrated that lower absolute GLS, GCS, and GRS were associated with increased incidence of heart failure, myocardial infarction, stroke, and death. All strain markers were independent predictors (incrementally above some respective conventional LV imaging markers) for the morbidity outcomes, but only GLS predicted death independently: (HR: 1.18; 95% CI: 1.07-1.30).

**Conclusions:**

In the general population, LV strain metrics derived using CMR-FT in radial, circumferential, and longitudinal directions are strongly and independently predictive of heart failure, myocardial infarction, and stroke, but only GLS is independently predictive of death in an adult population cohort.

Ventricular myocardial strain is presented as a percentage change in length when tracking the deformation of myocardium from the original length, which is commonly defined as the end-diastolic phase.^1^ It is traditionally measured with 3 different markers that reflect the contraction of the left ventricle (LV) along the longitudinal, radial, and circumferential axes.^2^ There is considerable interest in these markers, inasmuch as they have been shown to be more sensitive markers of cardiac dysfunction than LV ejection fraction (LVEF), which is widely used in the clinical setting,^3^, ^4^, ^5^ and multiple studies have demonstrated incremental diagnostic and prognostic value of strain over LVEF alone.^6^^,^^7^

The use of echocardiography-derived myocardial strain is well established in the clinical setting for detecting, monitoring, and planning interventions in various cardiac disease states.^8^^,^^9^ Cardiac magnetic resonance (CMR)-derived myocardial strain using feature or tissue tracking software has also shown promise in providing early diagnosis and prognostic value for conditions such as cardiomyopathies and myocarditis.^7^^,^^9^, ^10^, ^11^, ^12^, ^13^, ^14^ However, very few studies using echocardiography or CMR have demonstrated the prognostic value of myocardial strain in the general population. A systematic review has identified 8 echocardiography studies that have demonstrated prediction of adverse outcomes in the general population using speckle-tracking LV strain; most focus on global longitudinal strain (GLS), with only 1 investigating global circumferential strain (GCS) and global radial strain (GRS).^15^^,^^16^

CMR feature tracking (CMR-FT), also known as tissue tracking, is now widely used in clinical and research settings. FT uses balanced steady-state free precession short-axis (SAX) and long-axis (LAX) cine images and applies postprocessing “trackers” to features identified within the defined myocardium (within the epicardial and endocardial contours).^9^^,^^17^ Various software algorithms are available that then track the features through the cardiac phases, usually starting with LV end-diastole as the reference phase.

Studies in cohorts with specific cardiac diseases have shown the prognostic value of CMR-FT–derived myocardial strain. One study has demonstrated that CMR-FT adds incremental prognostic value above the use of clinical features, LVEF, and presence of late gadolinium enhancement (LGE) in patients with myocarditis.^7^ Another study conducted in >3,000 participants in the MESA (Multi-Ethnic Study of Atherosclerosis) has shown the LV GCS is a sensitive marker to differentiate between individuals with cardiovascular risk factors (obesity, smoking, hypertension, diabetes, and hypercholesterolemia) and those without.^18^ Another MESA study demonstrated the incremental prognostic value of CMR-derived GCS in predicting incident HF and cardiovascular events.^19^ Only 1 study using CMR tagging (another method of calculating strain) in an unselected cohort demonstrated the incremental prognostic value (in addition to LVEF and LGE) of LV GCS in predicting a composite cardiac endpoint.^6^ In summary, no prior studies have investigated the prognostic value of all 3 CMR-derived myocardial strain metrics such as GLS, GCS, and GRS (by any method) in a general population for individual cardiovascular endpoints and death.

This study aimed to investigate the prognostic value of CMR-FT–derived LV GLS, LV GCS, and LV GRS in the general population. We addressed the existing knowledge gap in the literature by assessing the predictive value of these strain metrics for heart failure (HF), myocardial infarction (MI), stroke, and death. Finally, we evaluated the incremental prognostic value of the strain markers over and above existing imaging markers known to be highly predictive of the respective outcomes being assessed.

## Methods

### Study population and design

The UK Biobank recruited participants aged 40 to 69 years between 2006 and 2010, with data collected on approximately 500,000 volunteers. Data collected include detailed sociodemographic and lifestyle information, physical measures, and blood samples.^20^ Longitudinal data on health outcomes was recorded using linkage to Hospital Episode Statistics and death registers. Further details on the UK Biobank protocol are publicly available.^21^ Additionally, approximately 50,000 CMR scans were performed (2015-2020) as part of the UK Biobank imaging study, which aims to perform imaging on ≤100,000 of the recruited participants.^22^

There were 45,893 scans available at the time of image analysis; however, 193 scans did not have any corresponding data output because of missing image sequences or corrupted files. Therefore, the study population consisted of the 45,700 participants whose scans were analyzable to derive strain data.

The endpoints of incident HF, incident MI, incident stroke, and death were defined using the Hospital Episodes Statistics data as detailed in Supplemental Table 1, where the dates recorded informed the censor dates. These endpoints and dates were derived for the whole study population, which included all participants who underwent CMR at the first imaging visit as part of the imaging study.

This study complies with the Declaration of Helsinki; the work was covered by the ethical approval for UK Biobank studies from the NHS National Research Ethics Service on 17th June 2011 (Ref 11/NW/0382) and extended on 18th June 2021 (Ref 21/NW/0157) with written informed consent obtained from all participants.

### CMR acquisition, image analysis, and quality control

Imaging in UK Biobank is performed with standardized staff training and equipment in specified centers. CMR is performed with 1.5-T scanners (MAGNETOM Aera, Syngo Platform VD13A, Siemens Healthcare). Cine images are with balanced steady-state free precession sequences. The UKB CMR protocol has been described in a dedicated publication.^23^

The CMR-FT strain analysis for the 45,700 scans available at the time of analysis was generated using automated batch processing developed by Circle Cardiovascular Imaging, Inc (cvi42 prototype 5.13.7). Automated contours were first drawn for SAX and LAX cine images with segmentation required only to define LV end diastole and LV end systole. Temporal smoothing was applied to the cines, with LV diastole (used as reference phase) and systole used to guide the feature tracking. Any SAX images with open LV contours were excluded from the feature tracking. The strain was calculated using a 2D algorithm that calculates myocardial deformation using a nearly incompressible deformation model,^24^^,^^25^ which acquires data from trackers placed within the segmented myocardium. The measurements of deformation are made in the radial, circumferential (all SAX slices), and longitudinal directions (all LAX cines). Various strain measurements were derived from the image analysis, but for the purposes of this report, we focus on peak GLS, GCS, and GRS, which were calculated by averaging the peak measurements across all myocardial points from all relevant slices that contained the tracked deformation. The strain module did not produce GCS and GRS in 4,390 scans (as a result of failed segmentation) and were therefore missing, but values were calculated for GLS in all 45,700 scans.

Quality control of the data output was performed using the removal of statistical outliers, which are 3 times the IQR (3 × IQR) below the first quartile and above the third quartile. We also removed non-sensical values defined as any GLS and GCS values >0 as well as any GRS values <0 because these are by default due to errors in the software tracking. This resulted in the exclusion of 244, 140, and 41 GLS, GCS, and GRS values (in addition to 4,390 missing values for GCS and GRS), respectively, out of 45,700. Before this approach, visual quality control was performed in 1,957 scans analyzed using the same batch processing method. The visual quality control demonstrated that 97% of the analyzed scans were “good.” There was no meaningful difference in derived imaging parameters when the output data from visual quality control and statistical outlier removal methods were compared. This supported our decision to use the latter for quality control across the entire data set.

Image analysis to derive volumetric data was performed using convolutional networks with a quality control process that has been described elsewhere.^26^ The left atrial volumes were derived using cvi42 (Circle Cardiovascular Imaging, Inc, prototype 5.11.0). The quality control of the atrial volumes included removing statistical outliers (3 × IQR).

Given the variety of automated image analysis pipelines and subsequent quality control used to generate the imaging markers of interest, there is variability in the availability of the necessary data.

### Statistical analysis

Analysis was systematically completed for each outcome (HF, MI, stroke, and death) and for each strain marker (LV GLS, LV GCS, LV GRS).

A univariable analysis was first carried out to demonstrate whether there was a significant association between each outcome and each strain marker, which were divided by quartiles. The association between each strain marker and each of the endpoints was then assessed using multivariable Cox regression models. The first models tested for all the strain markers and for all the endpoints included clinical covariates of interest, which were age, sex, ethnicity, body mass index (BMI), smoking and alcohol use, diabetes mellitus, prevalent hypertension, hypercholesterolemia, and prevalent coronary disease (the latter was excluded in models for MI as the endpoint). The subsequent models included the clinical covariates, the strain marker of interest, and additional imaging marker(s) that have been shown to provide significant prognostic or predictive value for the respective endpoint being tested. Left ventricular global function index (LVGFI), LVEDV, and LVEF were chosen as the imaging markers against which to test the incremental value of strain metrics for HF outcome. They had all been shown to have either significant predictive value (LVGFI)^27^^,^^28^ or wide clinical use (LVEDV and LVEF)^3^^,^^29^ for HF. Left ventricular end-systolic volume (LVESV), which had been shown to have significant prognostic value,^30^ LVEDV, and LVEF were chosen for the MI outcome. LVEDV and LVEF, despite their limitations, are widely used for predicting cardiovascular outcomes and are readily available markers.^3^^,^^29^ In addition, a secondary analysis was performed for the HF and MI outcomes to assess whether mitral annular plane systolic excursion (MAPSE) provides any additional prognostic benefit in addition to strain markers, which has been suggested in the literature.^31^, ^32^, ^33^ Left atrial (LA) maximal volume, shown to have significant predictive value for stroke,^34^^,^^35^ was chosen for the stroke outcome. Finally, LV mass, which had been shown to be significantly predictive of all-cause death,^36^^,^^37^ was chosen as the convention imaging marker to test against the death outcome. Incremental value of strain markers was tested using concordance statistics (C-statistics). All models used a complete-case only approach (see Supplemental Table 2 for sample size and number of values excluded for each model) and were tested for violation of the proportional hazards assumption. Multiple testing correction was applied to the multivariable results using the Bonferroni method (0.05/30 = 0.002).

All analyses were performed with R statistical software (v4.3.1, R Core Team 2023)^38^ and Python 3.9.16. The R packages used for statistical analysis are “survival,” “survminer,” “Hmisc,” and “survAUC” packages. Figures in results were produced using "ggplot2" and “forestplot” packages. The Python package used was “scikit-survival.”

## Results

### Baseline characteristics

The baseline characteristics of the study population are presented in Table 1. The UK Biobank field IDs used to define these characteristics can be found in Supplemental Table 3. The average age of the population was 55 years, with an equal distribution of men and women. The majority of our study cohort were White and nonsmokers. The average values of CMR parameters were within normal limits for the study population.^39^^,^^40^ The average LV GLS and GCS were lower (less negative) than the published normal average values for CMR-FT studies but not below the published cutoff value of −14%.^39^^,^^40^ There were no comparable or large studies that have reported average values for LV GRS in the general population, but the average in this study population (31%) was lower than the average reported for echocardiography measurements of LV GRS using 3D speckle tracking (45%).^41^

**Table 1:** Participant Characteristics (N = 45,700)

Demographics	
Age at baseline imaging, y	65 ± 7.7
Male/female, %	48/52
Ethnicity	
White	43,894 (96)
Other	1,806 (4)
Lifestyle factors	
Smoking	
Current	3,148 (7)
Previous	15,406 (34)
Never	26,808 (59)
BMI, kg/m^2^	26 (23.6-28.8)
Medical background	
Coronary artery disease	2,130 (5)
Hypercholesterolemia	11,453 (25)
Hypertension	13,354 (29)
Diabetes	2,200 (5)
CMR parameters	
Left ventricle	
Ejection fraction, %	60 ± 6.2
End-diastolic volume, mL	147 ± 33.8
End-systolic volume, mL	87 ± 19.4
Mass, g	86 ± 22.4
Left ventricle strain parameters	
Global longitudinal strain, %	−18 ± 2.3
Global circumferential strain, %	−19 ± 2.3
Global radial strain, %	31 ± 6.2
Right ventricle	
Ejection fraction, %	57 ± 6.2
End-diastolic volume, mL	156 ± 37.0
End-systolic volume, mL	67 ± 21.3
Left atrium	
Emptying fraction, %	66 ± 10.2
End-diastolic volume, mL	26 ± 13.6
End-systolic volume, mL	73 ± 25.8
Right atrium	
Emptying fraction, %	51 ± 10.8
End-diastolic volume, mL	40 ± 16.7
End-systolic volume, mL	82 ± 28.8

BMI = body mass index; CMR = cardiac magnetic resonance.

Values are mean ± SD, n (%), or median (Q1-Q3), unless otherwise indicated.

### Prognostic value of LV strain markers in predicting HF

**Figure 1:**
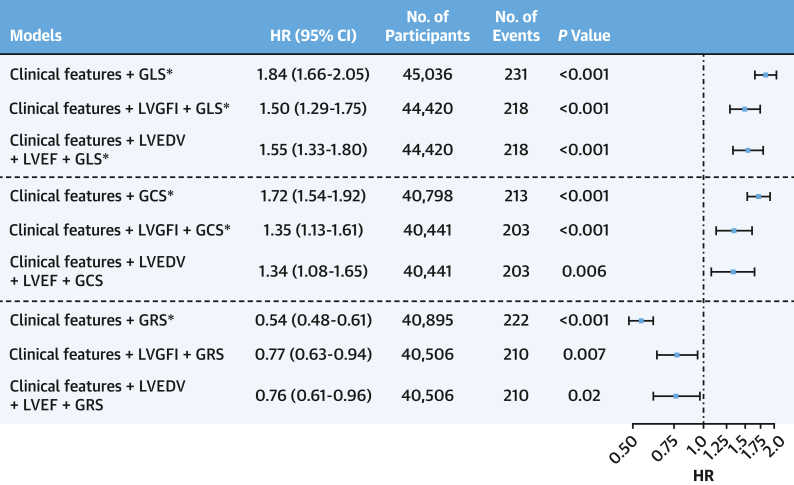
Global Myocardial Strain Predicts Heart Failure Clinical features included in all models were age, ethnicity, sex, smoking and alcohol status, body mass index, diabetes status, prevalent coronary disease, hypertension, and hypercholesterolemia. There was no violation of the proportional hazard assumption in all models. ∗Indicates model significant after multiple testing correction using the Bonferroni method. LV GCS = left ventricular global circumferential strain (%); LV GLS = left ventricular global longitudinal strain (%); LV GRS = left ventricular global radial strain (%); LVEDV = left ventricular end-diastolic volume (mL), LVEF = left ventricular ejection fraction (%); LVGFI = left ventricular global function index.

All 3 strain markers were predictive of HF in the univariable analysis (Supplemental Figures 1 to 3). All 3 strain markers were independent predictors of HF in fully adjusted multivariable Cox regression models (HR: 1.84; 95% CI: 1.66-2.05; HR: 1.72; 95% CI: 1.54-1.92; HR: 0.54; 95% CI: 0.48-0.61) for LV GLS, GCS, and GRS, respectively), as well as in models additionally adjusted for conventional imaging markers (Figure 1). Additionally, all 3 strain markers provided incremental prognostic value, as indicated by higher C-statistics, over and above clinical features (Supplemental Table 4). All LV GLS models were still significant after applying multiple testing correction. Additionally, LV GCS models that included clinical features and LVGFI, and LV GRS model including clinical features, remained significant after applying multiple testing correction. In the secondary analysis, CMR-FT strain metrics outperformed MAPSE by retaining independent prognostic associations (Supplemental Table 5).

### Prognostic value of LV strain markers in predicting MI

**Figure 2:**
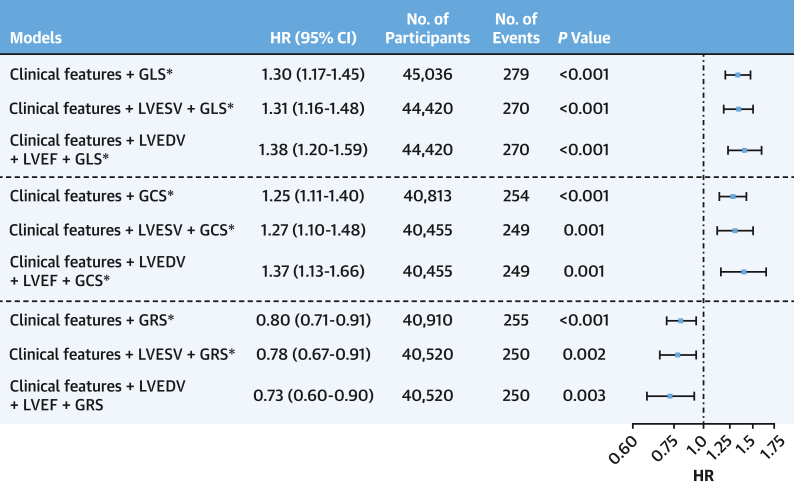
Global Myocardial Strain Predicts Myocardial Infarction Clinical features included in all models were age, ethnicity, sex, smoking and alcohol status, body mass index, diabetes status, hypertension, and hypercholesterolemia. There was no violation of the proportional hazard assumption in all models. ∗Indicates model significant after multiple testing correction using the Bonferroni method. LVESV = left ventricular end-systolic volume; other abbreviations as in Figure 1.

All 3 strain markers were predictive of MI in the univariable analysis (Supplemental Figures 4 to 6). In fully adjusted multivariable Cox regression models, all 3 strain markers provided independent prognostic value (HR: 1.30; 95% CI: 1.17-1.45; HR: 1.25; 95% CI: 1.11-1.40; and HR: 0.80 95% CI: 0.71-0.91 for LV GLS, GCS, and GRS, respectively), as well as in models with additional imaging markers (Figure 2). Additionally, GLS provided incremental prognostic value in a model with LVESV, and both GLS and GCS provided incremental value in a model with LVEDV and LVEF (Supplemental Table 6). All models except GRS with clinical features and LVEDV and LVEF were significant after applying multiple testing correction.

### Prognostic value of LV strain markers in predicting stroke

**Figure 3:**
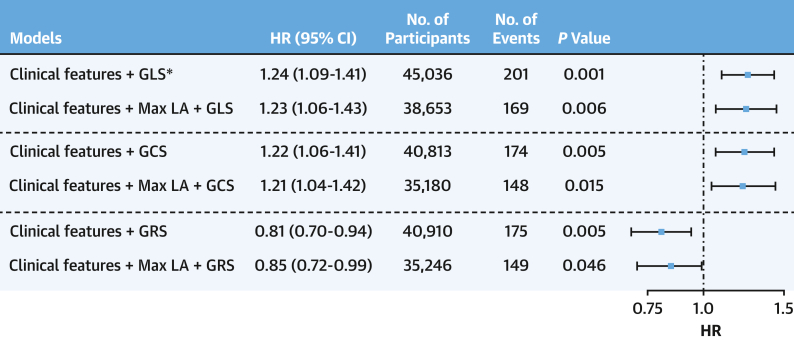
Global Myocardial Strain Predicts Stroke Clinical features included in all models were age, ethnicity, sex, smoking and alcohol status, body mass index, diabetes status, prevalent coronary disease, hypertension, and hypercholesterolemia. There was no violation of the proportional hazard assumption in all models. ∗Indicates model significant after multiple testing correction using the Bonferroni method. Max LA = maximum left atrial volume (mL); other abbreviations as in Figure 1.

All 3 strain markers were predictive of stroke in the univariable analysis (Supplemental Figures 7 to 9). In fully adjusted multivariable models, all 3 strain markers were independent predictors of stroke (HR: 1.24; 95% CI: 1.09-1.41; HR: 1.22 05% CI: 1.06-1.41; HR: 0.81; 95% CI: 0.70-0.94 for LV GLS, GCS, and GRS, respectively). The global strain markers also provided independent value even after the inclusion of LA maximum volume in the model (HR: 1.23; 95% CI: 1.06-1.43; HR: 1.21; 95% CI: 1.04-1.42; HR: 0.85; 95% CI: 0.72-0.99 for LV GLS, GCS, and GRS respectively) (Figure 3). Only 1 model with LV GLS and clinical features was significant after applying multiple testing correction, and the model C-statistic analysis did not demonstrate incremental value (Supplemental Table 7).

### Prognostic value of LV strain markers in predicting death

**Figure 4:**
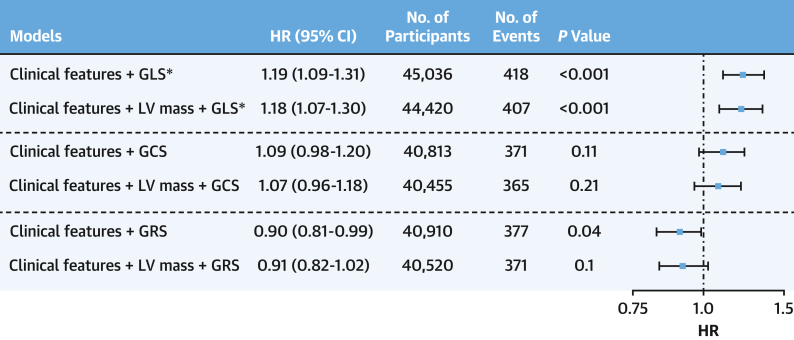
Global Myocardial Strain Predictive Value for Death Clinical features included in all models were age, ethnicity, sex, smoking and alcohol status, body mass index, diabetes status, prevalent coronary disease, hypertension, and hypercholesterolemia. There was no violation of the proportional hazard assumption in all models. ∗Indicates model significant after multiple testing correction using the Bonferroni method. LV mass = left ventricular mass (g); other abbreviations as in Figure 1.

All 3 strain markers were predictive of death in the univariable analysis (Supplemental Figures 10 to 12). However, only GLS provided independent prognostic value above clinical features (HR: 1.19; 95% CI: 1.09-1.31) and LV mass (HR: 1.18; 95% CI: 1.07-1.30) (Figure 4). GRS provided marginal independent prognostic value above clinical features (HR: 0.90; 95% CI: 0.81-0.99; *P* = 0.04) but not when LV mass was also included in the model. Only GLS models remained significant after applying multiple testing correction, and none provided evidence of incremental value (Supplemental Table 8).

## Discussion

This unique study investigated the predictive and prognostic value of CMR-FT–generated strain measurements in a large general population. The prevalence of diabetes, coronary artery disease, and other cardiovascular risk factors in the study population was comparable with that in the general population (Supplemental Table 9).^42^^,^^43^ However, the proportion of smokers^43^ and participants from other ethnic minority backgrounds was lower in the study population than in the general population,^44^ with the average BMI lower than the average for this age group in the general population.^45^ All 3 CMR myocardial strain markers (LV GLS, LV GCS, LV GRS), demonstrated predictive value for HF, MI, stroke, and death in univariable analyses, as summarized in the Central Illustration. These strain markers also provided independent predictive value when adjusted for clinical covariates for heart failure, MI, and stroke. These results are comparable with those of other population studies that have assessed the prognostic value of myocardial strain. As an important novel finding, our study also demonstrated incremental prognostic values of GLS and GCS over well-established imaging markers for HF and MI. LVGFI, LVEDV, and LVEF have been shown to be very good indicators of cardiac dysfunction resulting in HF, but the results have demonstrated that GLS and GCS can provide additional prognostic information. Similarly, LVESV, LVEDV, and LVEF have been shown to be very good indicators of poor prognosis associated with MI, but the results demonstrate the GLS and GCS can provide additional prognostic information.

**Central Illustration:**
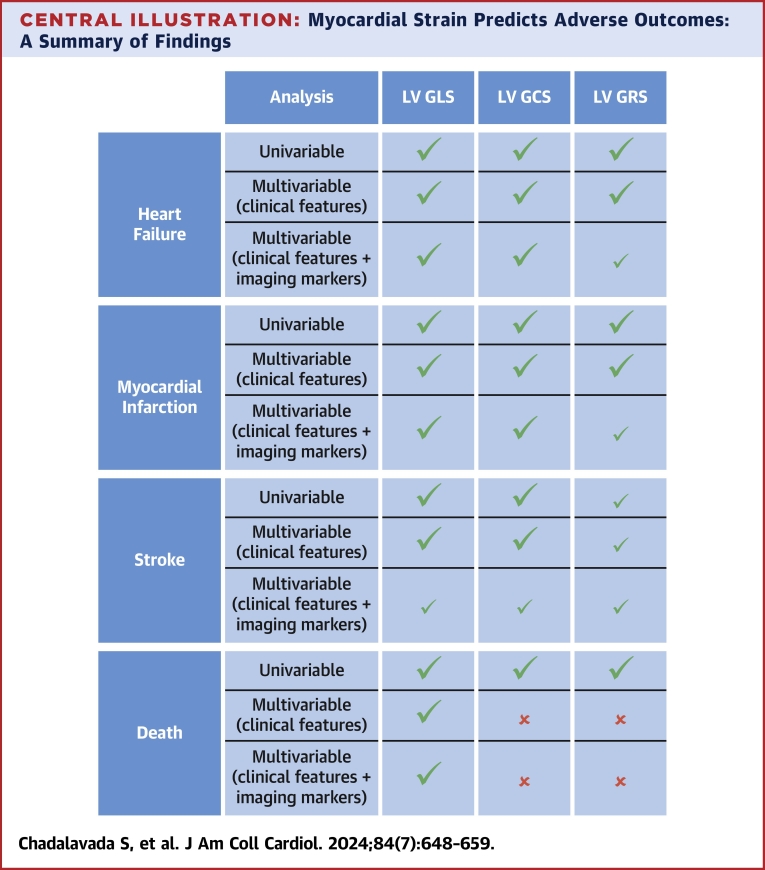
Myocardial Strain Predicts Adverse Outcomes: A Summary of Findings Green checkmark indicates that the strain marker showed prognostic value for that model and the *P* value was <0.05. If green checkmark is bold, then the models remained significant after adjusting for multiple testing correction using the Bonferroni method. Red x indicates that the result was statistically insignificant. Clinical features included in the multivariable models were age, sex, body mass index, ethnicity, smoking and alcohol use, diabetes, hypertension, and coronary disease (except in myocardial infarction models). LV GCS = left ventricular global circumferential strain; LV GLS = left ventricular global longitudinal strain; LV GRS = left ventricular global radial strain.

Furthermore, we showed that the strain markers provided independent predictive value for adverse outcomes even after accounting for demographics, cardiovascular risk factors, and other established prognostic imaging measurements. Uniquely, our study demonstrated these results in 3 separate components of myocardial strain, not just LV GLS, which is the most studied strain parameter in the literature. Most of the association results remained significant (especially for HF and MI) even after applying the strictest multiple testing correction using the Bonferroni method.

Our findings demonstrated that CMR myocardial strain using feature tracking is highly sensitive to cardiac dysfunction and superior to current commonly used imaging markers in a community-based low-risk population. LV GLS and LV GRS predicted all-cause death in a multivariable model adjusted for clinical covariates. LV GLS independently predicted death after adjusting for LV mass. It also showed an incremental value over clinical covariates that are established predictors of death. The LV GCS and GRS results were not as promising, with either borderline or nonsignificant results for all-cause mortality. This finding is expected, because LV GCS and GRS are perhaps more specific to cardiac-specific adverse outcomes reflecting dysfunction in the mid and epicardial layers.^46^ In studies using speckle tracking echocardiography, LV GLS has been shown to predict death in the general population.^15^^,^^47^ One study has demonstrated the incremental benefit of CMR-FT–generated LV GLS over LVEF and LGE in predicting death in those with dilatated cardiomyopathy.^48^ Our study showed an added prognostic value of CMR-generated LV GLS independently of LV mass for all-cause mortality in a general population.

In clinical practice and in research, echocardiography techniques using Doppler imaging or speckle tracking are leading the way in evaluating myocardial strain. These are associated with lower costs and greater accessibility compared with CMR imaging. However, a significant limitation is that the reliability of the data is heavily influenced by the acquisition of good-quality images, which can be patient- and operator dependent. CMR images are not impeded by the limitation of good acoustic windows and have been shown to have superior reproducibility with less operator dependence compared with echocardiography.^49^

### Study strengths and limitations

This study addresses a gap in current knowledge by investigating and demonstrating the prognostic value of CMR-FT myocardial strain measurements in a large general population. One key advantage of the feature tracking method is its applicability in a standard CMR study without the need for additional dedicated image sequences. Given that feature tracking can be applied in noncontrast medium cine images, important and prognostic information can still be inferred in those patients with contraindications to the use of contrast material.

Specific benefits are offered by the software used in this study, which was developed by Circle Cardiovascular Imaging, Inc (cvi42). It uses automated algorithms to define end systole and end diastole, and also to define the endocardial and epicardial borders, which reduces any interoperator variability, which was previously quoted to be a limitation of feature tracking software.^50^ Given that cvi42 is widely used in clinical settings, integration of fully automated strain module can be relatively straightforward.

However, clinicians still need to be aware of the limitations of the software. Poorly aligned cine images, for example, can result in erroneous measurements. Additionally, there may be specific conditions in which using feature tracking would not be appropriate. For example, in apical hypertrophic cardiomyopathy, because of the cavity obliteration, the feature tracking software could not effectively track the features that disappear between phases, hence producing inappropriate results. Another limitation of this study is that the findings are yet to be validated within other populations and clinical cohorts. This limitation presents opportunities for future research in diverse cohorts. Last, this study included the UK Biobank population, which is known to have a healthy-volunteer selection bias. However, this reinforces the message that strain measurements are powerful even in a relatively low-risk population, as demonstrated in a previous study in the same population.^51^

### Clinical perspectives

The findings of this study highlight the potential of CMR-FT myocardial strain for widespread clinical applications, with the capability to provide more sensitive and earlier prognostic data in disease states. This would be a major advantage in monitoring and potentially guiding treatment in a wide range of cardiac conditions such as cardiomyopathies and infiltrative conditions such as amyloidosis and myocarditis. It could also transform practices in subspecialities like cardio-oncology, where currently LVEF is used as a crude marker to continue or halt treatments. To expedite the use of CMR-derived myocardial strain in clinical practice, normal values and cutoff points need to be better established. However, this endeavor has been limited by the variability in published reference ranges and cutoff points derived from various competing methods and software algorithms.^2^^,^^39^^,^^40^ Therefore, the comparability of these values is questionable. This study is the first to demonstrate in a large data set, at a population level, the prognostic value of the CMR myocardial strain derive by a regulatory compliant, commercial software provider. Once reference ranges are established, the prognostic value of a normal CMR, which has already been demonstrated in the literature,^52^, ^53^, ^54^ would be further enhanced if the scan also had normal myocardial strain measurements.

## Conclusions

LV GLS, LV GCS, and LV GRS generated using CMR-FT software are valuable imaging markers that are predictive of HF, MI, stroke, and death. They provide incremental value over and above existing well-established imaging markers. Further research should be conducted to validate the findings of this study in diverse populations to pave the way toward clinical integration.

Perspectives **COMPETENCY IN PATIENT CARE AND PROCEDURAL SKILLS:** Myocardial strain measured by CMR identifies subclinical cardiac dysfunction that is associated with adverse cardiovascular outcomes.

**TRANSLATIONAL OUTLOOK:** Further research is needed to define and standardize normal ranges and disease-specific and risk factor–specific thresholds for CMR-derived myocardial train to facilitate broad clinical use.

## Funding Support and Author Disclosures

This work acknowledges the support of the National Institute for Health and Care Research Barts Biomedical Research Centre (NIHR203330); a delivery partnership of Barts Health NHS Trust, Queen Mary University of London, St George’s University Hospitals NHS Foundation Trust and St George’s University of London. Barts Charity (G-002346) contributed to fees required to access UK Biobank data [access application #2964]. This paper is supported by the London Medical Imaging and Artificial Intelligence Centre for Value Based Healthcare (AI4VBH), which is funded from the Data to Early Diagnosis and Precision Medicine strand of the government’s Industrial Strategy Challenge Fund, managed and delivered by Innovate UK on behalf of UK Research and Innovation (UKRI). Views expressed are those of the authors and not necessarily those of the AI4VBH Consortium members, the NHS, Innovate UK, or UKRI. The funders did not have any role in the study design, data collection and analysis, decision to publish, or preparation of the manuscript. Dr Petersen acknowledges the British Heart Foundation for funding the manual analysis to create a cardiovascular magnetic resonance imaging reference standard for the UK Biobank imaging resource in 5,000 CMR scans (PG/14/89/31194). Dr Aung acknowledges the Medical Research Council for supporting his Clinician Scientist Fellowship (MR/X020924/1). Dr Chadalavada was funded by European Union's Horizon 2020 research and innovation program under grant agreement no. 825903 (euCanSHare project). Dr Rauseo is supported by the mini-Centre for Doctoral Training (CDT) award through the Faculty of Science and Engineering, Queen Mary University of London, United Kingdom. Dr Naderi was supported by the British Heart Foundation Pat Merriman Clinical Research Training Fellowship (FS/20/22/34640). Dr Petersen and Dr Lee acknowledge support from the SmartHeart EPSRC program grant (EP/P001009/1) and the European Union's Horizon 2020 research and innovation program under grant agreement No 825903 (euCanSHare project). Dr Petersen has served as a consultant for Cardiovascular Imaging Inc, Calgary, Alberta, Canada. All other authors have reported that they have no relationships relevant to the contents of this paper to disclose.
