# Supplementary material for: Myocardial Strain Measured by Cardiac Magnetic Resonance Predicts Cardiovascular Morbidity and Death
Source: J Am Coll Cardiol. 2024 Aug 13;84(7):648–59. doi: 10.1016/j.jacc.2024.05.050 (PMC11320766; doi:10.1016/j.jacc.2024.05.050)
Supplement: Supplemental Tables 1-9 and Supplemental Figures 1-12 [file mmc1.docx]

**Myocardial Strain Measured by Magnetic Resonance Imaging Predicts Cardiovascular Morbidity and Death**

**SUPPLEMENTAL MATERIAL**

[Supplemental Table 1 - Defining outcomes of interest 2](#_Toc163489165)

[Supplemental Table 2 - Sample size and number of participants excluded from respective regression models 3](#_Toc163489166)

[Supplemental Table 3 - Defining co-variates of interest 5](#_Toc163489167)

[Supplemental Table 4 - C-statistics for heart failure models 6](#_Toc163489168)

[Supplemental Table 5 - Predictive value of strain markers vs MAPSE 7](#_Toc163489169)

[Supplemental Table 6 - C-statistics for myocardial infarction models 8](#_Toc163489170)

[Supplemental Table 7- C-statistics for stroke models 9](#_Toc163489171)

[Supplemental Table 8- C-statistics for death models 10](#_Toc163489172)

[Supplemental Table 9 - Comparing results to other population studies of myocardial strain 11](#_Toc163489173)

[Supplemental Figure 1 - Longitudinal strain predicts heart failure 12](#_Toc162813094)

[Supplemental Figure 2 - Circumferential strain predicts heart failure 13](#_Toc162813095)

[Supplemental Figure 3 - Radial strain predicts heart failure 14](#_Toc162813096)

[Supplemental Figure 4 - Longitudinal strain predicts myocardial infarction 15](#_Toc162813097)

[Supplemental Figure 5 - Circumferential strain predicts myocardial infarction 16](#_Toc162813098)

[Supplemental Figure 6 - Radial strain predicts myocardial infarction 17](#_Toc162813099)

[Supplemental Figure 7 - Longitudinal strain predicts stroke 18](#_Toc162813100)

[Supplemental Figure 8 - Circumferential strain predicts stroke 19](#_Toc162813101)

[Supplemental Figure 9 - Radial strain predicts stroke 20](#_Toc162813102)

[Supplemental Figure 10 - Longitudinal strain predicts death 21](#_Toc162813103)

[Supplemental Figure 11 - Circumferential strain predicts death 22](#_Toc162813104)

[Supplemental Figure 12 - Radial strain predicts death 23](#_Toc162813105)

Supplemental Table 1 - Defining outcomes of interest

| Outcome | UKBB data field(s) | Code |
| --- | --- | --- |
| Heart failure | 41270  41280 | I500, I501, I509, I110, I130, I132, J81 |
| Myocardial infarction | 41270  41280 | I21, I210, I211, I212, I213, I214, I219, I21X, I22, I220, I221, I228, I229, I23, I230, I231, I232, I233, I234, I235, I236, I238, I24, I248, I249 |
| Stroke | 41270  41280 | I630, I631, I632, I633, I634, I635, I636, I638, I639, I64, I650, I651, I652, I653, I658, I659, I660, I661, I662, I663, I664, I668, I669, I693 |
| Death | 4000 | Date death occurred |

Supplemental Table 2 - Sample size and number of participants excluded from respective regression models

| Cox regression model | Sample size | No of participants excluded |
| --- | --- | --- |
| Heart failure | | |
| Clinical features + GLS | 45036 | 664 |
| Clinical features + LVGFI +GLS | 44420 | 1280 |
| Clinical features + LVEDV + LVEF + GLS | 44420 | 1280 |
| Clinical features + GCS | 40798 | 4902 |
| Clinical features + LVGFI + GCS | 40441 | 5259 |
| Clinical features + LVEDV + LVEF + GCS | 40441 | 5259 |
| Clinical features + GRS | 40895 | 4805 |
| Clinical features + LVGFI + GRS | 40506 | 5194 |
| Clinical features + LVEDV + LVEF + GRS | 40506 | 5194 |
| Myocardial infarction | | |
| Clinical features + GLS | 45036 | 3974 |
| Clinical features + LVESV + GLS | 44420 | 4590 |
| Clinical features + LVEDV + LVEF + GLS | 44420 | 4590 |
| Clinical features + GCS | 40813 | 8197 |
| Clinical features + LVESV + GCS | 40455 | 8555 |
| Clinical features + LVEDV + LVEF + GCS | 40455 | 8555 |
| Clinical features + GRS | 40910 | 255 |
| Clinical features + LVESV + GRS | 40520 | 8490 |
| Clinical features + LVEDV + LVEF + GRS | 40520 | 8490 |
| Stroke | | |
| Clinical features + GLS | 45036 | 3977 |
| Clinical features + LA max + GLS | 38653 | 10360 |
| Clinical features + GCS | 40813 | 174 |
| Clinical features + LA max + GCS | 35180 | 13833 |
| Clinical features + GRS | 40910 | 8103 |
| Clinical features + LA max + GRS | 35246 | 13676 |
| Death | | |
| Clinical features + GLS | 45036 | 3977 |
| Clinical features + LVM + GLS | 44420 | 4593 |
| Clinical features + GCS | 40813 | 8200 |
| Clinical features + LVM + GCS | 40555 | 8558 |
| Clinical features + GRS | 40910 | 8103 |
| Clinical features + LVM + GRS | 40520 | 8493 |
| GLS – Global longitudinal strain, LVGFI – Left ventricular global function index, LVEDV – Left ventricular end diastolic volume, LVEF – Left ventricular ejection fraction, GCS – Global circumferential strain , LVESV – Left ventricular end systolic volume, GRS –Global radial strain, LA max – Left atrium maximum volume, LVM – LV mass | | |

Supplemental Table 3 - Defining co-variates of interest

| **Covariate** | **UKBB data field(s)** | **Code** |
| --- | --- | --- |
| Age | 21003 (at imaging visit) | - |
| Sex | 31 | - |
| BMI | 21001 | - |
| Ethnicity group | 21000 | - |
| Smoking | 20116 (smoking status)  20160 (Ever smoked) | - |
| Alcohol | 20117 (Alcohol drinker status) | - |
| Diabetes | Self-report (20002) | type 1 diabetes  type 2 diabetes |
|  | Diagnosed with Diabetes (2443) | Yes |
|  | Medications (2003) | Metformin  Gliclazide  Glimepiride  Glipizide  Tolbutamide  Pioglitazone  Insulin product  Repaglinide  Neteglinide |
|  | Insulin use in last year (2986) | Yes |
| Type 1 diabetes | Self-report (20002) | type 1 diabetes |
|  | ICD10 (130706) | E10 (date first reported) |
| Type 2 diabetes | Self-report (20002) | type 2 diabetes |
|  | ICD10 (130708) | E11 (date first reported) |
| Prevalent heart failure | Self-report (2002) | Heart failure/ pulmonary oedema |
|  | ICD10 (131354) | I50 |
| Prevalent hypertension | Self-report (20002) | hypertension  essential hypertension |
|  | Medication for cholesterol, blood pressure or diabetes (6177) | Blood pressure medication |
| High cholesterol | Self-report (20002) | high cholesterol |
|  | Medication for cholesterol, blood pressure or diabetes (6177) | 1, Cholesterol lowering medication |
| Prevalent Coronary disease | Self-report (20002) | heart attack/myocardial infarction |
|  | ICD10 (131298, 131300, 131302, 131304, 131306) | I20, I22, I23, I24, I25 (Date first reported) |
|  | Coronary procedures (41272) | K40, K41, K42, K43, K44, K45, K46, K47, K48, K49, K50, K75 |
| Prevalent stroke | Self-report (2002) | Transient ischaemic attack  Ischaemic stroke |
|  | ICD10 (131366, 131368, 131370, 131372, 131374, 131376, 131378) | I63, I64, I65, I66, I67, I68 I69  (Date first reported) |

Supplemental Table 4 - C-statistics for heart failure models

| Models | Harrel’s C-statistic [95% CI] | P value | Uno C-statistic [95% CI] | P value |
| --- | --- | --- | --- | --- |
| Clinical features | 0.80 [0.76 to 0.82] | <0.001 | 0.73 [0.61 to 0.82] | <0.001 |
| Clinical features + GLS | 0.82 [0.79 to 0.85] |  | 0.81 [0.73 to 0.87] |  |
|  | | | | |
| Clinical features + LVGFI | 0.82 [0.80 to 0.85] | 0.06 | 0.78 [0.65 to 0.87] | 0.001 |
| Clinical features +LVGFI + GLS | 0.82 [0.80 to 0.86] |  | 0.81 [0.71 to 0.88] |  |
|  | | | | |
| Clinical features + LVEDV + LVEF | 0.84 [0.81 to 0.86] | 0.05 | 0.77 [0.62 to 0.88] | <0.001 |
| Clinical features + LVEDV + LVEF + GLS | 0.84 [0.81 to 0.87] |  | 0.80 [0.66 to 0.90] |  |
|  | | | | |
| Clinical features | 0.79 [0.76 to 0.82] | <0.001 | 0.74 [0.63 to 0.83] | <0.001 |
| Clinical features + GCS | 0.82 [0.80 to 0.85] |  | 0.81 [0.69 to 0.90] |  |
|  | | | | |
| Clinical features + LVGFI | 0.82 [0.79 to 0.85] | 0.04 | 0.77 [0.64 to 0.88] | 0.007 |
| Clinical features +LVGFI + GCS | 0.83 [0.79 to 0.86] |  | 0.80 [0.66 to 0.90] |  |
|  | | | | |
| Clinical features + LVEDV + LVEF | 0.84 [0.80 to 0.86] | 0.93 | 0.77 [0.60 to 0.89] | 0.09 |
| Clinical features + LVEDV + LVEF + GCS | 0.83 [0.80 to 0.86] |  | 0.79 [0.63 to 0.90] |  |
|  | | | | |
| Clinical features | 0.80 [0.76 to 0.82] | <0.001 | 0.73 [0.64 to 0.84] | <0.001 |
| Clinical features + GRS | 0.82 [0.79 to 0.85] |  | 0.80 [0.67 to 0.89] |  |
|  | | | | |
| Clinical features + LVGFI | 0.83 [0.80 to 0.86] | 0.30 | 0.78 [0.65 to 0.88] | 0.40 |
| Clinical features +LVGFI + GRS | 0.83 [0.80 to 0.86] |  | 0.79 [0.63 to 0.90] |  |
|  | | | | |
| Clinical features + LVEDV + LVEF | 0.84 [0.82 to 0.87] | 0.20 | 0.76 [0.62 to 0.88] | 0.28 |
| Clinical features + LVEDV + LVEF + GRS | 0.84 [0.81 to 0.87] |  | 0.77 [0.62 to 0.91] |  |
| Clinical features adjusted for were: age, ethnicity, sex, smoking and alcohol status, body mass index, diabetes status, prevalent coronary disease, hypertension and hypercholesterolaemia.  GLS – Global longitudinal strain, LVGFI – Left ventricular global function index, LVEDV – Left ventricular end diastolic volume, LVEF – Left ventricular ejection fraction, GCS – Global circumferential strain , GRS –Global radial strain. | | | | |

Supplemental Table 5 - Predictive value of strain markers vs MAPSE

| Model | HR for MAPSE [95% CI] | P value | HR for strain marker [95% CI] | P value |
| --- | --- | --- | --- | --- |
| Clinical features + MAPSE | 0.64 [0.57 – 0.72] | <0.001 | n/a | - |
| Clinical features+ GLS + MAPSE | 0.91 [0.79 – 1.05] | 0.19 | 1.77 [1.57 – 2.01] | <0.001 |
|  | | | | |
| Clinical features + MAPSE | 0.64 [0.57 – 0.72] | <0.001 | n/a | - |
| Clinical features+ GCS + MAPSE | 0.79 [0.69 – 0.91] | <0.001 | 1.59 [1.41 – 1.79] | <0.001 |
|  | | | | |
| Clinical features + MAPSE | 0.64 [0.57 – 0.72] | <0.001 | n/a | - |
| Clinical features + GRS + MAPSE | 0.79 [0.69 – 0.90] | <0.001 | 0.60 [0.53 – 0.68] | <0.001 |
| Clinical features + MAPSE | 0.93[0.83 – 1.04] | 0.25 | n/a | - |
| Clinical features+ GLS + MAPSE | 1.02[0.90 – 1.16] | <0.001 | 1.26[1.11 – 1.42] | <0.001 |
|  | | | | |
| Clinical features + MAPSE | 0.93[0.83 – 1.04] | 0.25 | n/a | - |
| Clinical features+ GCS + MAPSE | 0.94[0.83 – 1.07] | 0.34 | 1.16[1.03 – 1.30] | 0.02 |
|  | | | | |
| Clinical features + MAPSE | 0.93[0.83 – 1.04] | 0.25 | n/a | - |
| Clinical features + GRS + MAPSE | 0.94[0.83 – 1.07] | 0.36 | 0.86 [0.76 – 0.98] | 0.02 |
| Clinical features adjusted for were: age, ethnicity, sex, smoking and alcohol status, body mass index, diabetes status, prevalent coronary disease (except for in myocardial infarction models), hypertension and hypercholesterolaemia.  GLS – Global longitudinal strain, MAPSE – Mitral annular plane systolic excrusion, GCS – Global circumferential strain , GRS –Global radial strain. | | | | |

Supplemental Table 6 - C-statistics for myocardial infarction models

| Models | Harrel’s C-statistic [95% CI] | P value | Uno C-statistic | P value |
| --- | --- | --- | --- | --- |
| Clinical features | 0.70 [0.68 to 0.73] | <0.001 | 0.68 [0.55 to 0.77] | 0.26 |
| Clinical features + GLS | 0.72 [0.70 to 0.74] |  | 0.69 [0.57 to 0.79] |  |
|  | | | | |
| Clinical features + LVESV | 0.70 [0.68 to 0.73] | <0.001 | 0.66 [0.53 to 0.77] | 0.003 |
| Clinical features +LVESV + GLS | 0.71 [0.68 to 0.74] |  | 0.69 [0.57 to 0.79] |  |
|  | | | | |
| Clinical features + LVEDV + LVEF | 0.71 [0.68 to 0.73] | <0.001 | 0.66 [0.52 to 0.77] | <0.001 |
| Clinical features + LVEDV + LVEF + GLS | 0.72 [0.69 to 0.75] |  | 0.70 [0.60 to 0.77] |  |
|  | | | | |
| Clinical features | 0.70 [0.68 to 0.73] | <0.001 | 0.68 [0.53 to 0.80] | 0.04 |
| Clinical features + GCS | 0.71 [0.68 to 0.74] |  | 0.66 [0.50 to 0.82] |  |
|  | | | | |
| Clinical features + LVESV | 0.70 [0.67 to 0.73] | <0.001 | 0.66 [0.52 to 0.77] | 0.81 |
| Clinical features +LVESV + GCS | 0.71 [0.68 to 0.74] |  | 0.66 [0.50 to 0.79] |  |
|  | | | | |
| Clinical features + LVEDV + LVEF | 0.71 [0.68 to 0.73] | 0.003 | 0.65 [0.48 to 0.79] | 0.02 |
| Clinical features + LVEDV + LVEF + GCS | 0.71 [0.68 to 0.74] |  | 0.69 [0.56 to 0.83] |  |
|  | | | | |
| Clinical features | 0.70 [0.67 to 0.73] | <0.001 | 0.68 [0.55 to 0.77] | <0.001 |
| Clinical features + GRS | 0.71 [0.68 to 0.74] |  | 0.65 [0.49 to 0.78] |  |
|  | | | | |
| Clinical features + LVESV | 0.70 [0.67 to 0.73] | <0.001 | 0.67 [0.55 to 0.77] | 0.20 |
| Clinical features +LVESV + GRS | 0.71 [0.68 to 0.74] |  | 0.65 [0.46 to 0.79] |  |
|  | | | | |
| Clinical features + LVEDV + LVEF | 0.70 [0.66 to 0.74] | <0.001 | 0.67 [0.57 to 0.79] | 0.57 |
| Clinical features + LVEDV + LVEF + GRS | 0.71 [0.69 to 0.74] |  | 0.68 [0.54 to 0.81] |  |
| Clinical features adjusted for were: age, ethnicity, sex, smoking and alcohol status, body mass index, diabetes status, hypertension and hypercholesterolaemia.  GLS – Global longitudinal strain, LVEDV – Left ventricular end diastolic volume, LVEF – Left ventricular ejection fraction, GCS – Global circumferential strain , LVESV – Left ventricular end systolic volume, GRS –Global radial strain | | | | |

Supplemental Table 7- C-statistics for stroke models

| Models | Harrel’s C-statistic | P value | Uno C-statistic | P value |
| --- | --- | --- | --- | --- |
| Clinical features | 0.69 [0.65 to 0.72] | <0.001 | 0.70 [0.66 to 0.75] | 0.001 |
| Clinical features + GLS | 0.69 [0.66 to 0.73] |  | 0.71 [0.67 to 0.77] |  |
|  | | | | |
| Clinical features + LA max | 0.68 [0.64 to 0.72] | <0.001 | 0.71 [0.66 to 0.75] | 0.006 |
| Clinical features +LA max + GLS | 0.70 [0.66 to 0.72] |  | 0.72 [0.68 to 0.76] |  |
|  | | | | |
| Clinical features | 0.69 [0.64 to 0.73] | <0.001 | 0.70 [0.64 to 0.75] | 0.009 |
| Clinical features + GCS | 0.71 [0.67 to 0.74] |  | 0.71 [0.66 to 0.76] |  |
|  | | | | |
| Clinical features + LA max | 0.70 [0.67 to 0.74] | 0.008 | 0.71 [0.66 to 0.75] | 0.92 |
| Clinical features +LA max + GCS | 0.71 [0.66 to 0.74] |  | 0.71 [0.65 to 0.76] |  |
|  | | | | |
| Clinical features | 0.69 [0.65 to 0.73] | <0.001 | 0.69 [0.64 to 0.75] | 0.02 |
| Clinical features + GRS | 0.70 [0.67 to 0.73] |  | 0.70 [0.65 to 0.75] |  |
|  | | | | |
| Clinical features + LA max | 0.70 [0.66 to 0.74] | 0.61 | 0.70 [0.66 to 0.75] | 0.42 |
| Clinical features +LA max + GRS | 0.70 [0.67 to 0.74] |  | 0.71 [0.65 to 0.75] |  |
| Clinical features adjusted for were: age, ethnicity, sex, smoking and alcohol status, body mass index, diabetes status, hypertension, coronary disease and hypercholesterolaemia.  GLS – Global longitudinal strain, GCS – Global circumferential strain, GRS –Global radial strain, LA max – Left atrium maximum volume. | | | | |

Supplemental Table 8- C-statistics for death models

| Models | Harrel’s C-statistic | P value | Uno C-statistic | P value |
| --- | --- | --- | --- | --- |
| Clinical features | 0.72 [0.69 to 0.75] | 0.37 | 0.70 [0.59 to 0.79] | 0.02 |
| Clinical features + GLS | 0.72 [0.70 to 0.74] |  | 0.72 [0.60 to 0.82] |  |
|  | | | | |
| Clinical features + LVM | 0.72 [0.69 to 0.75] | 0.40 | 0.72 [0.58 to 0.83] | 0.97 |
| Clinical features +LVM + GLS | 0.72 [0.69 to 0.75] |  | 0.70 [0.59 to 0.79] |  |
| Clinical features adjusted for were: age, ethnicity, sex, smoking and alcohol status, body mass index, diabetes status, hypertension, coronary disease and hypercholesterolaemia.  GLS – Global longitudinal strain, GCS – Global circumferential strain, GRS –Global radial strain, LVM – LV mass | | | | |

Supplemental Table 9 - Comparing results to other population studies of myocardial strain

| Study | End point assessed | Imaging modality & Strain marker assessed | HR [95% CI], (p value) | Closest comparison model(s) from this study | HR [95% CI], (p value) |
| --- | --- | --- | --- | --- | --- |
| ^1^Mordi I et al. | MACE | CMR-FT GCS (+ clinical variables) | 1.22 [1.14 - 1.231], (p <0.001) | Clinical features + GCS (HF outcome) | 1.50 [1.29 – 1.75] |
|  |  |  |  | Clinical features + GCS (MI outcome) | 1.25[1.11 – 1.40] |
| ^1^Mordi I et al. | MACE | CMR-FT GCS (+ clinical variables + LGE and LVEF) | 1.11 [1.02 - 1.21], (p = 0.04) | Clinical features + GCS + LVEF + LVED (HF outcome) | 1.34 [1.08 + 1.65], (p = 0.006) |
|  |  |  |  | Clinical features + GCS + LVEF + LVED (MI outcome) | 1.37 [1.13 – 1.66], (p = 0.001) |
| ^2^Cheng S et al. | Coronary heart disease | Speckle echo – longitudinal strain + clinical features | 1.29 [1.00 -1.67], (p =0.05) | Clinical features + GLS (MI outcome) | 1.30 [1.17 – 1.45], (p<0.001) |
| ^2^Cheng S et al. | HF | Speckle echo – GCS + clinical features | 1.79 [1.35–2.37], (p <0.0001) | Clinical features + GCS (HF outcome) | 1.72 [1.54 – 1.92], (p<0.001) |
| ^3^Al Saikhan L et al. | Mortality | Speckle echo - GLS | 1.07 [1.03-1.11], (p = 0.001)** | Clinical features + GLS (death outcome) | 1.19 [1.09 – 1.31], (p<0.001) |
| ^3^Al Saikhan L et al. | Coronary heart disease | Speckle echo - GLS | 1.15 [1.03-1.29], (p = 0.017)** | Clinical features + GLS (for MI outcome) | 1.30 [1.17 – 1.45], (p<0.001) |
| ^4^ Choi et al | Heart failure | CMR-tagging GCS (+ clinical features + LVMi + LVEF | 1.40 [1.02 – 1.91], (p = 0.03)* | Clinical features + GCS (HF outcome) + LVEF + LVED | 1.34 [1.08 + 1.65], (p = 0.006) |
| ^1^Mordi I et al. The Combined Incremental Prognostic Value of LVEF, Late Gadolinium Enhancement, and Global Circumferential Strain Assessed by CMR. *JACC Cardiovasc Imaging*. 2015;8:540–549.v  ^2^Cheng S et al. Distinct Aspects of Left Ventricular Mechanical Function Are Differentially Associated With Cardiovascular Outcomes and All-Cause Mortality in the Community https://doi.org/10.1161/JAHA.115.002071.  ^3^Al Saikhan L et al. Prognostic implications of left ventricular strain by speckle-tracking echocardiography in population-based studies: a systematic review protocol of the published literature. *BMJ Open*. 2018;8:23346  ^4^ Choi EY, Rosen BD, Fernandes VRS, et al. Prognostic value of myocardial circumferential strain for incident heart failure and cardiovascular events in asymptomatic individuals: the Multi-Ethnic Study of Atherosclerosis. *Eur Heart J*. 2013;34:2354–2361.  MACE – Major adverse cardiac events, HF – Heart failure, CMR – Cardiac magnetic resonance, FT – Feature tracking, LVEF – Left ventricular ejection fraction, LGE – Late gadolinium enhancement, LVMi – LV mass indexed, GLS – Global longitudinal strain, GCS – Global circumferential strain, GRS – Global radial strain  *results changed from HR per 1% to per SD in order to make for closest comparison.  ** taken from metanalysis which included mix of results per SD and % change. | | | | | |

Supplemental Figure 1 - Longitudinal strain predicts heart failure


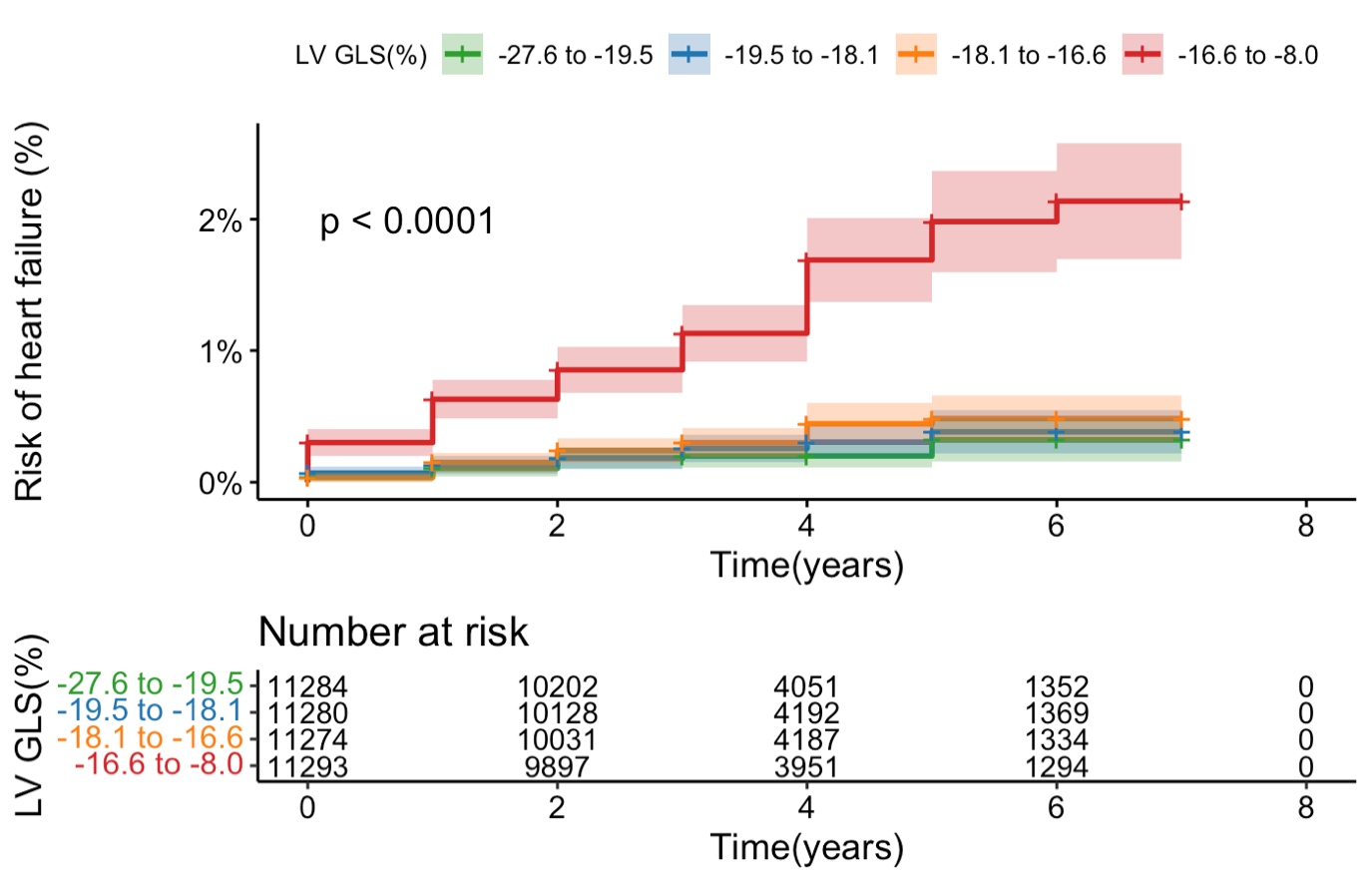


A survival plot from univariable analysis demonstrating increasing risk of heart failure associated with deteriorating left ventricular global longitudinal strain (LV GLS), shown divided by quartiles.

Supplemental Figure 2 - Circumferential strain predicts heart failure


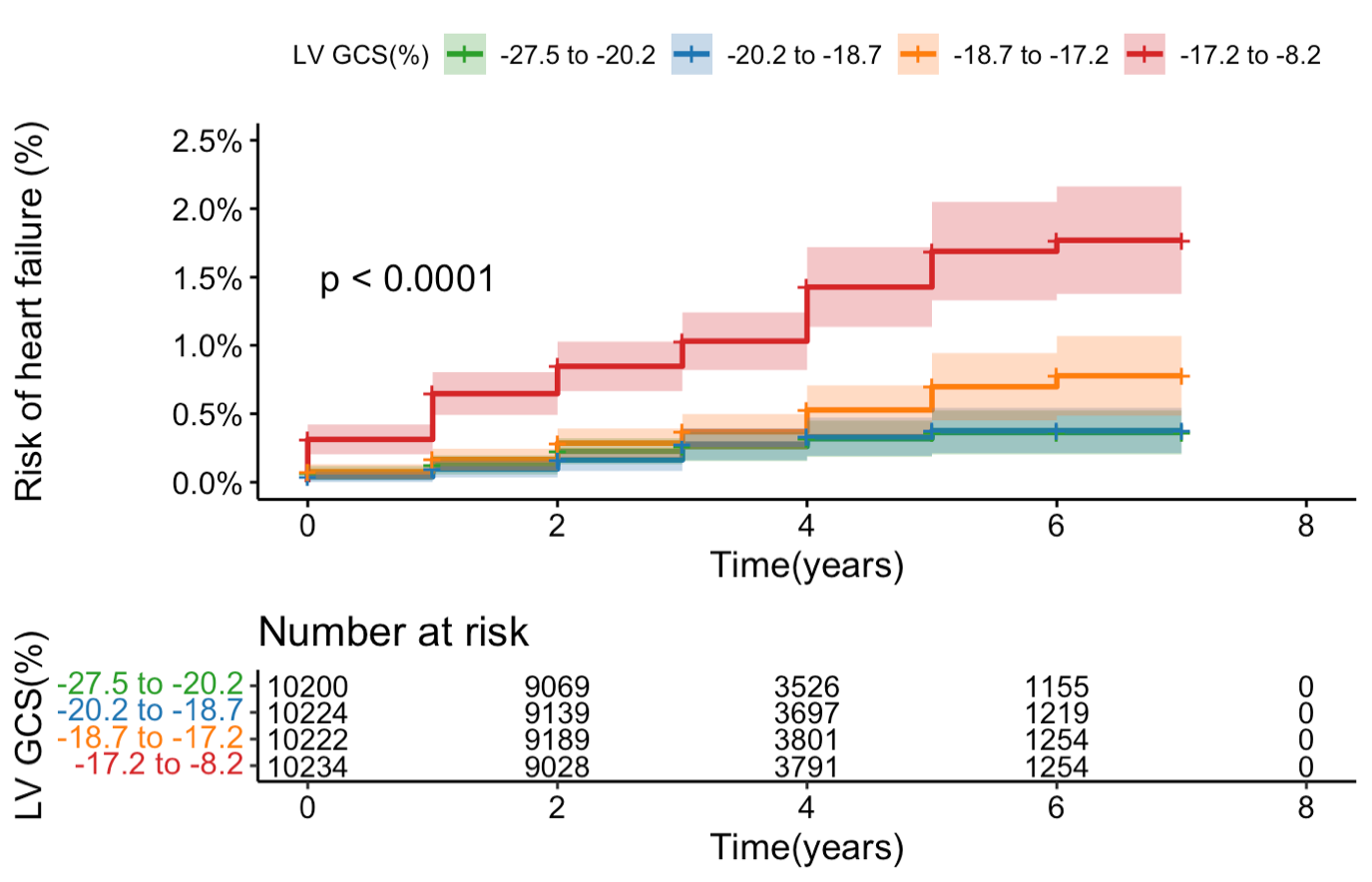


A survival plot from survival analysis demonstrating increased risk of heart failure associated with deteriorating left ventricular global circumferential strain (LV GCS), shown divided by quartiles.

Supplemental Figure 3 - Radial strain predicts heart failure


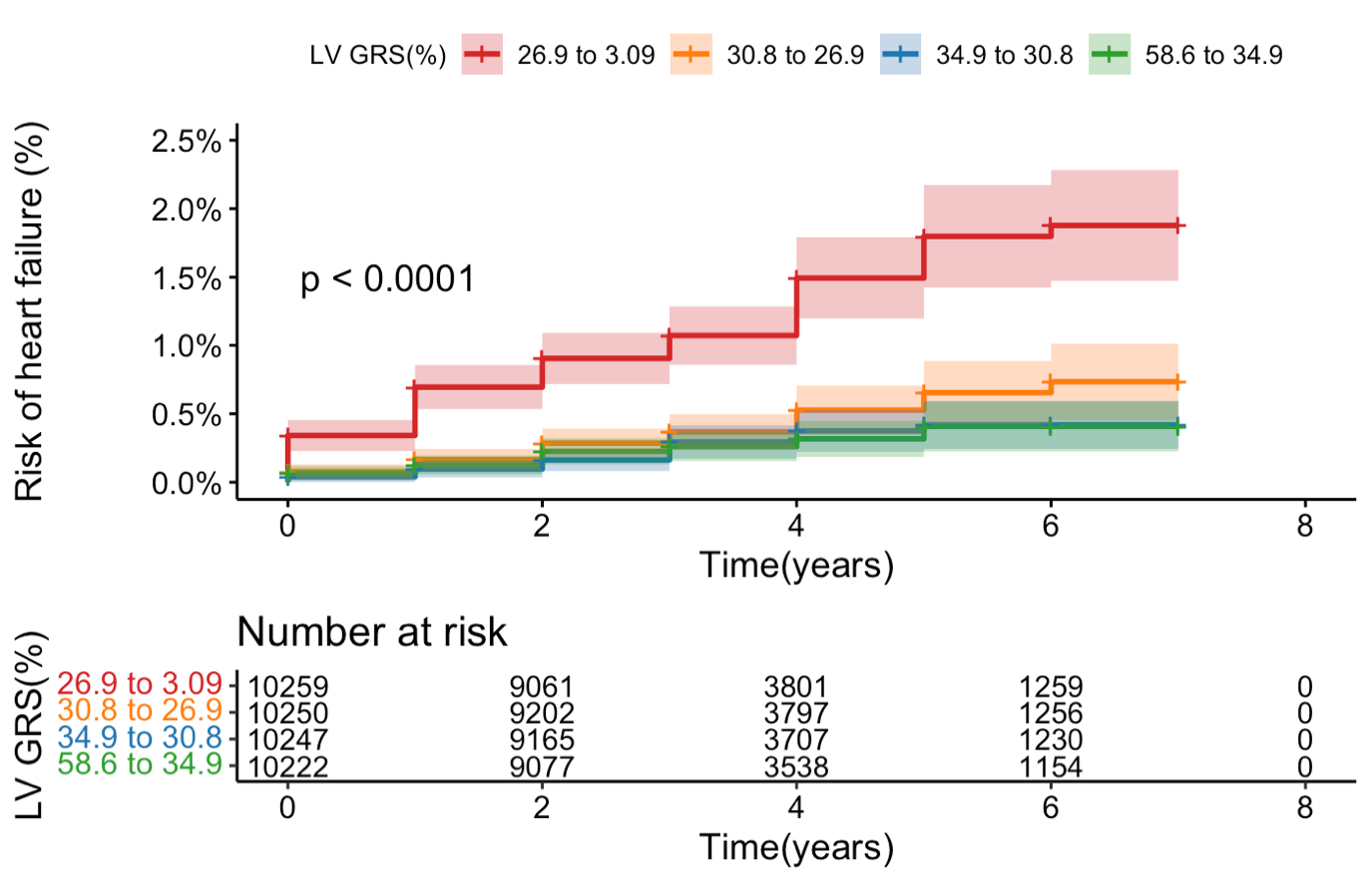


A survival plot from univariable analysis demonstrating increased risk of heart failure associated with deteriorating left ventricular global radial strain (LV GRS), shown divided by quartiles.

Supplemental Figure 4 - Longitudinal strain predicts myocardial infarction


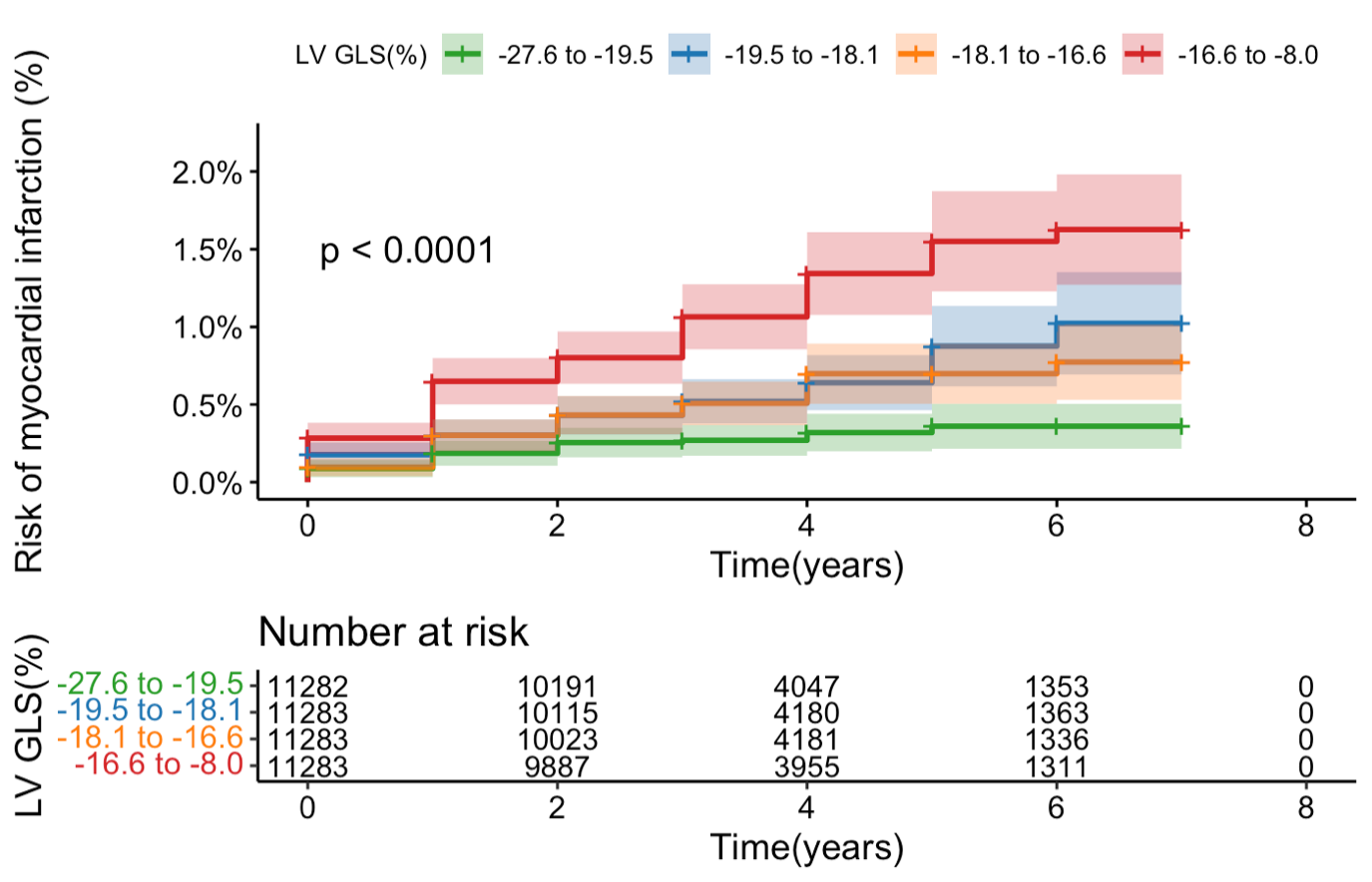


A survival plot from univariable analysis demonstrating increased risk of myocardial infarction associated with deteriorating left ventricular global longitudinal strain (LV GLS), shown divided by quartiles.

Supplemental Figure 5 - Circumferential strain predicts myocardial infarction


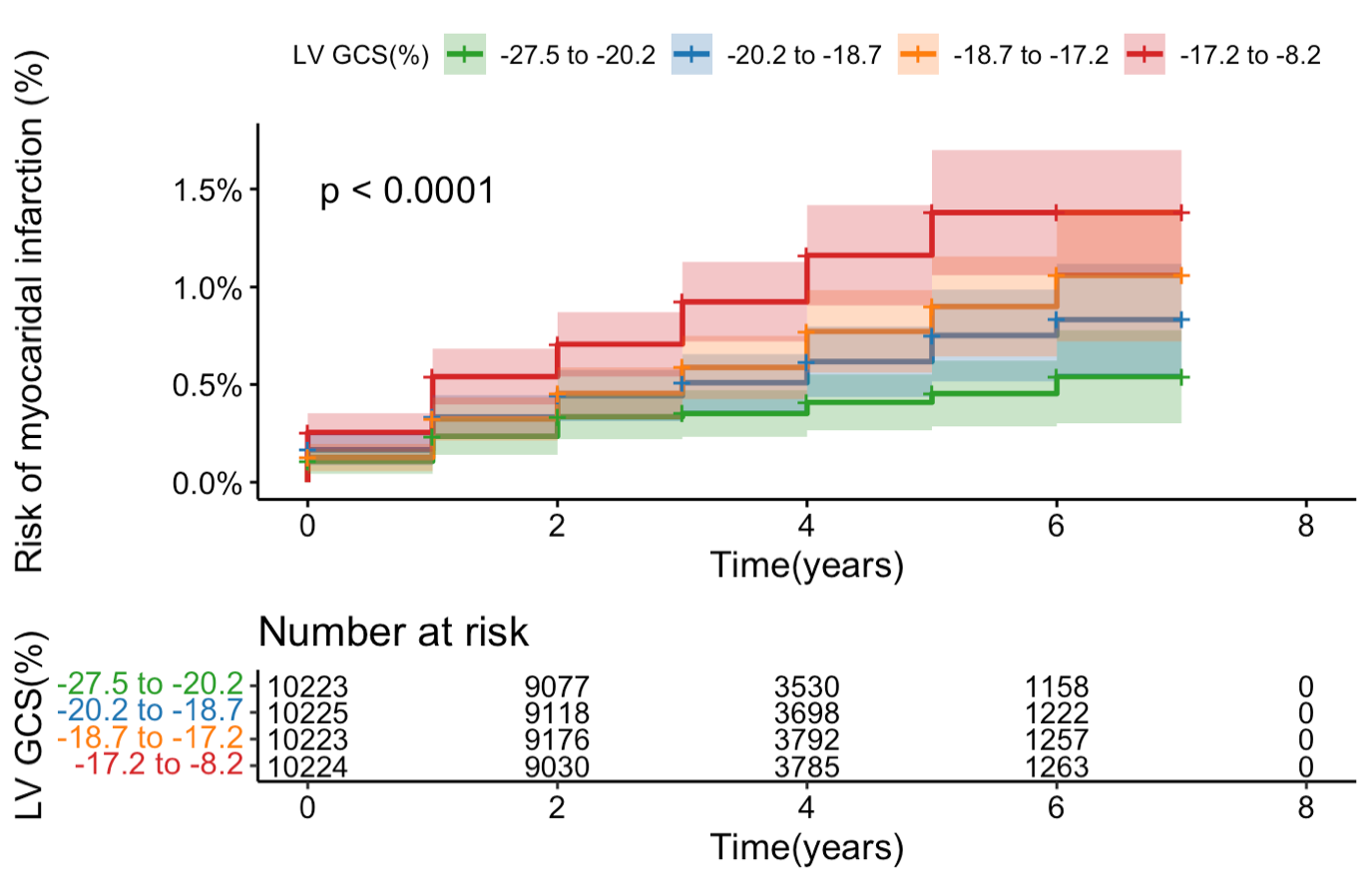


A survival plot from univariable analysis demonstrating increased risk of myocardial infarction associated with deteriorating left ventricular global circumferential strain deteriorates (LV GCS), shown divided by quartiles.

Supplemental Figure 6 - Radial strain predicts myocardial infarction


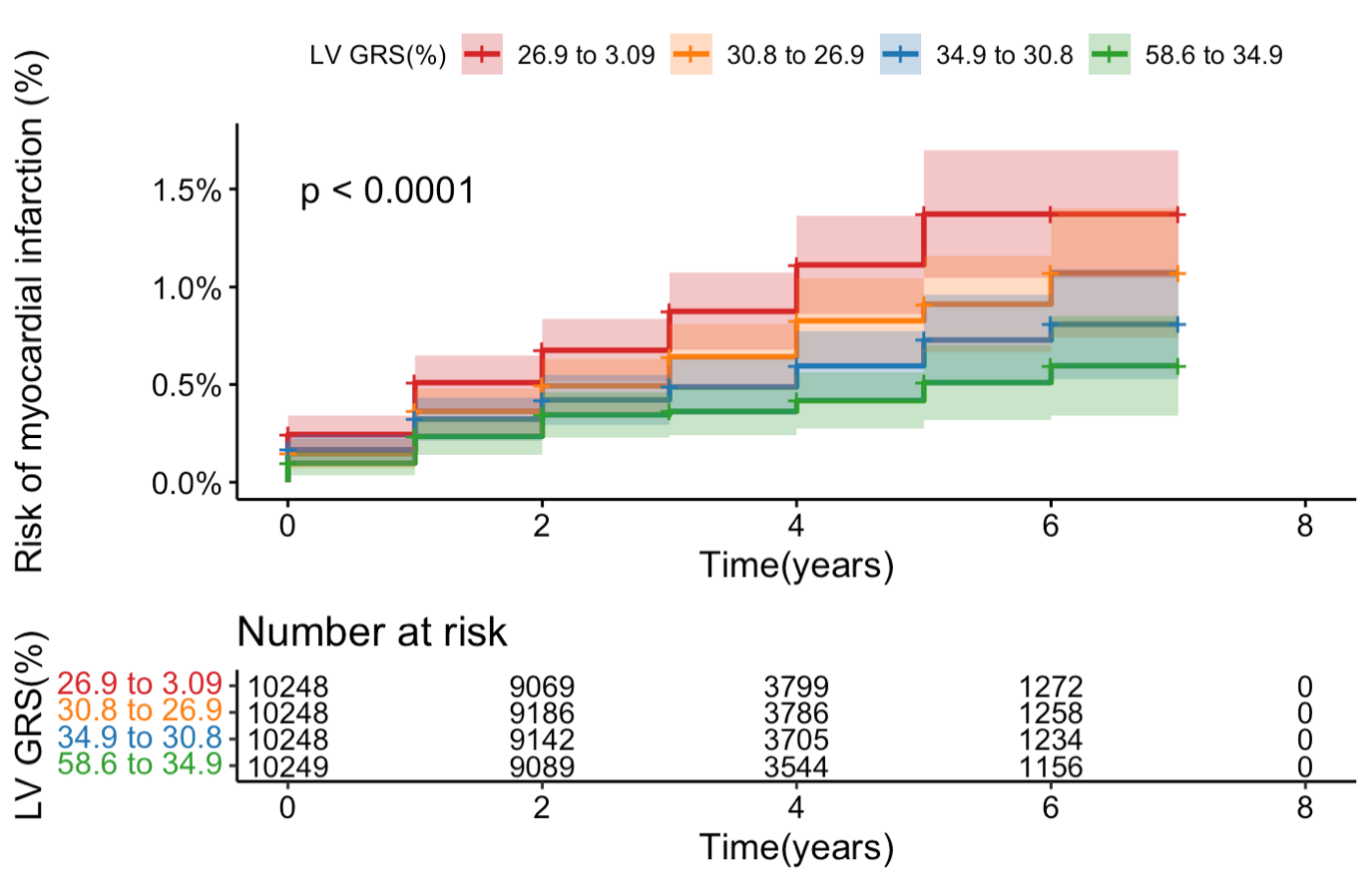


A survival plot from univariable analysis demonstrating increased risk of myocardial infarction associated with deteriorating left ventricular global radial strain deteriorates (LV GRS), shown divided by quartiles.

Supplemental Figure 7 - Longitudinal strain predicts stroke


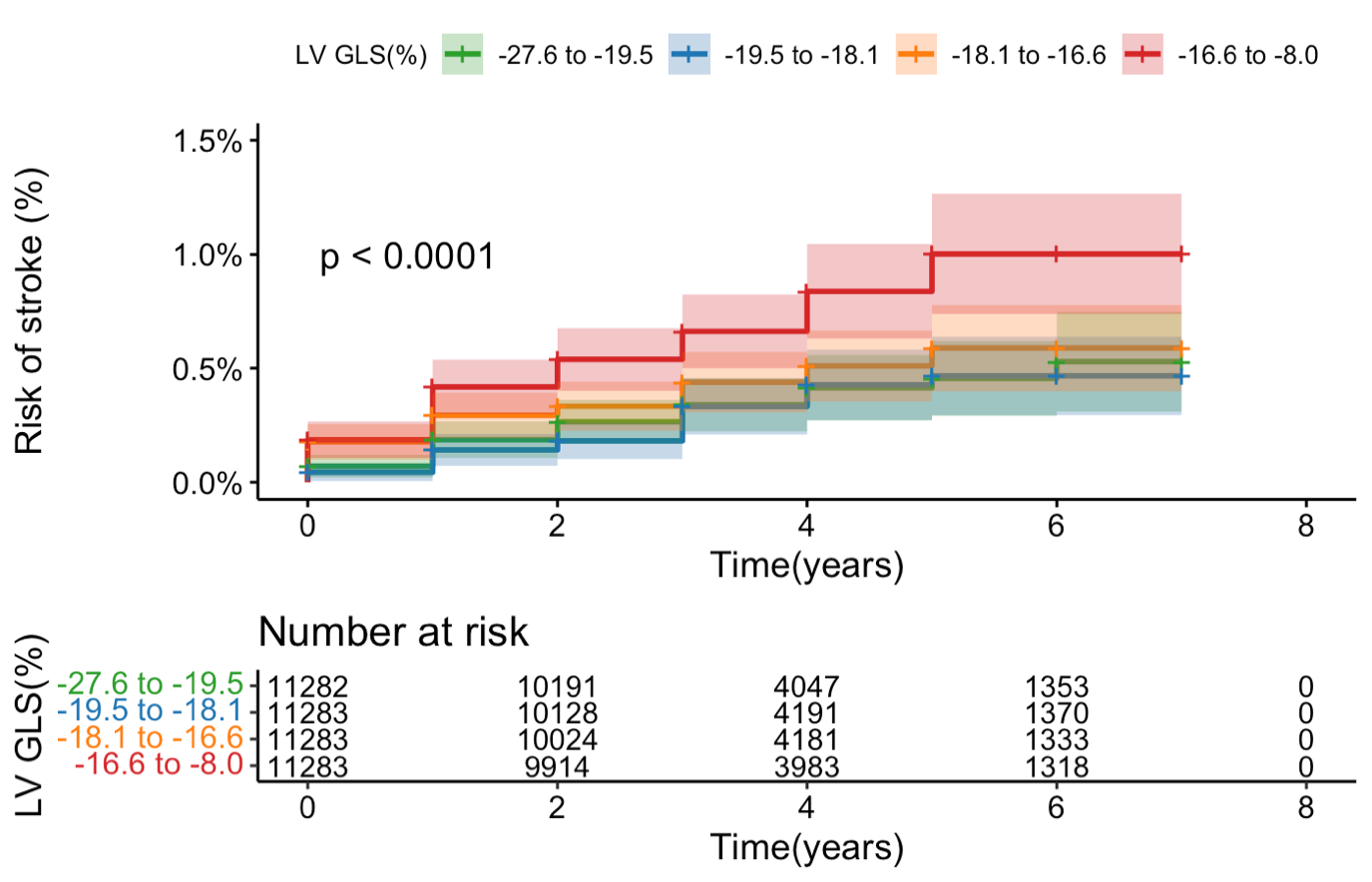


A survival plot from univariable analysis demonstrating increased risk of stroke associated with deteriorating left ventricular global longitudinal strain deteriorates (LV GLS), shown divided by quartiles.

Supplemental Figure 8 - Circumferential strain predicts stroke


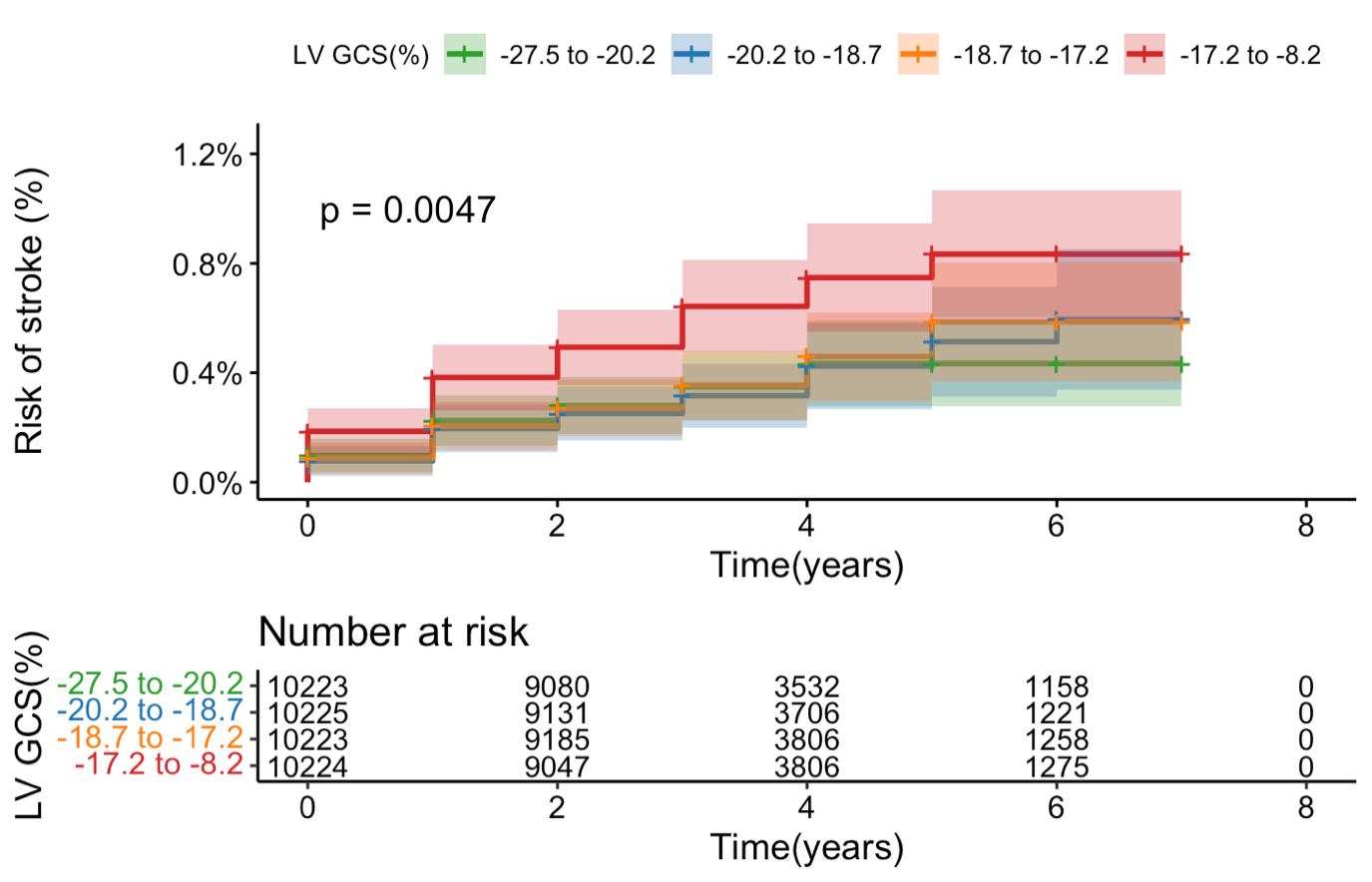


A survival plot from univariable analysis demonstrating increased risk of stroke associated with deteriorating left ventricular global circumferential strain deteriorates (LV GCS), shown divided by quartiles.

Supplemental Figure 9 - Radial strain predicts stroke


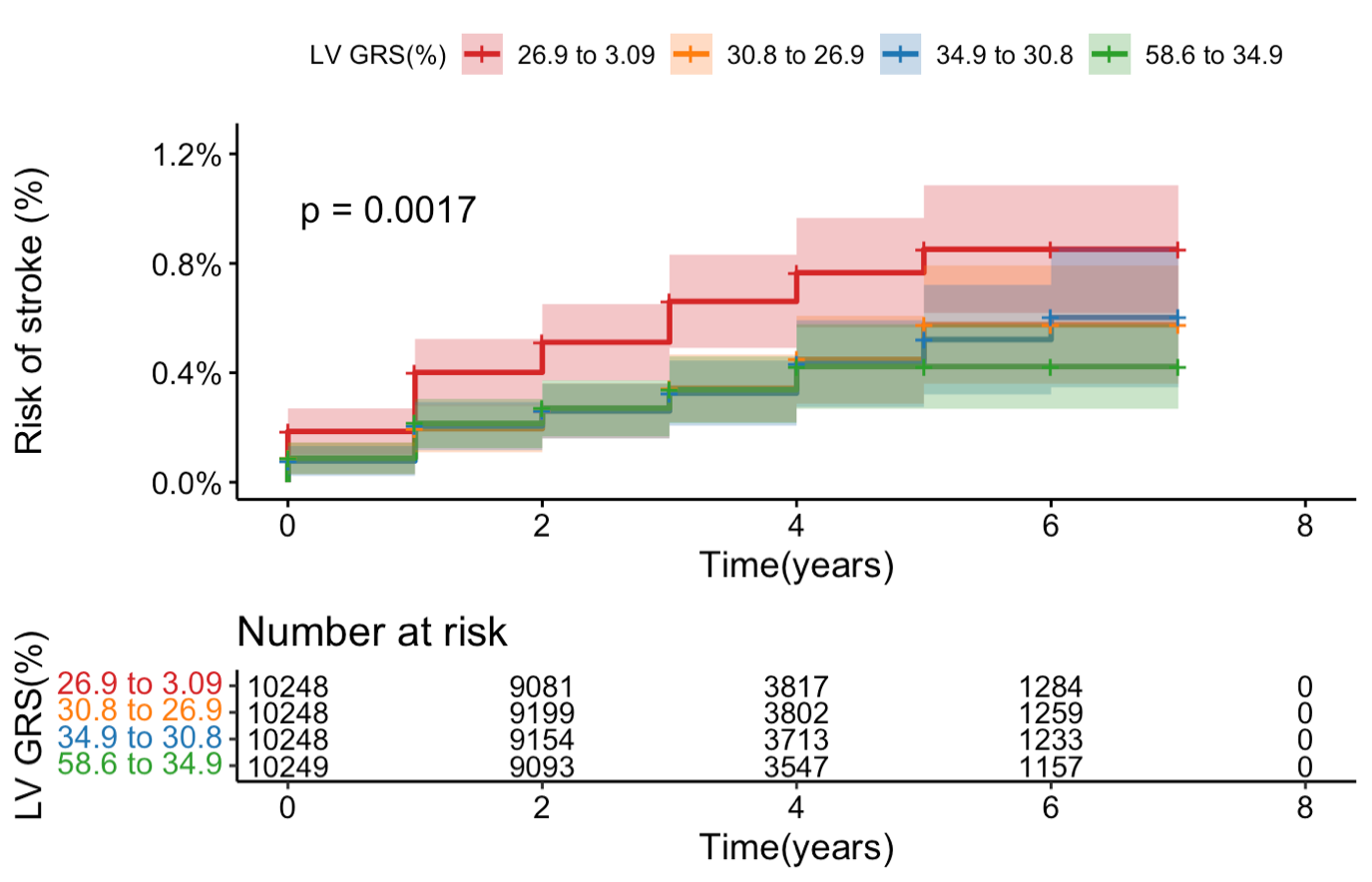


A survival plot from univariable analysis demonstrating increased risk of stroke associated with deteriorating left ventricular global radial strain deteriorates (LV GRS), shown divided by quartiles

Supplemental Figure 10 - Longitudinal strain predicts death


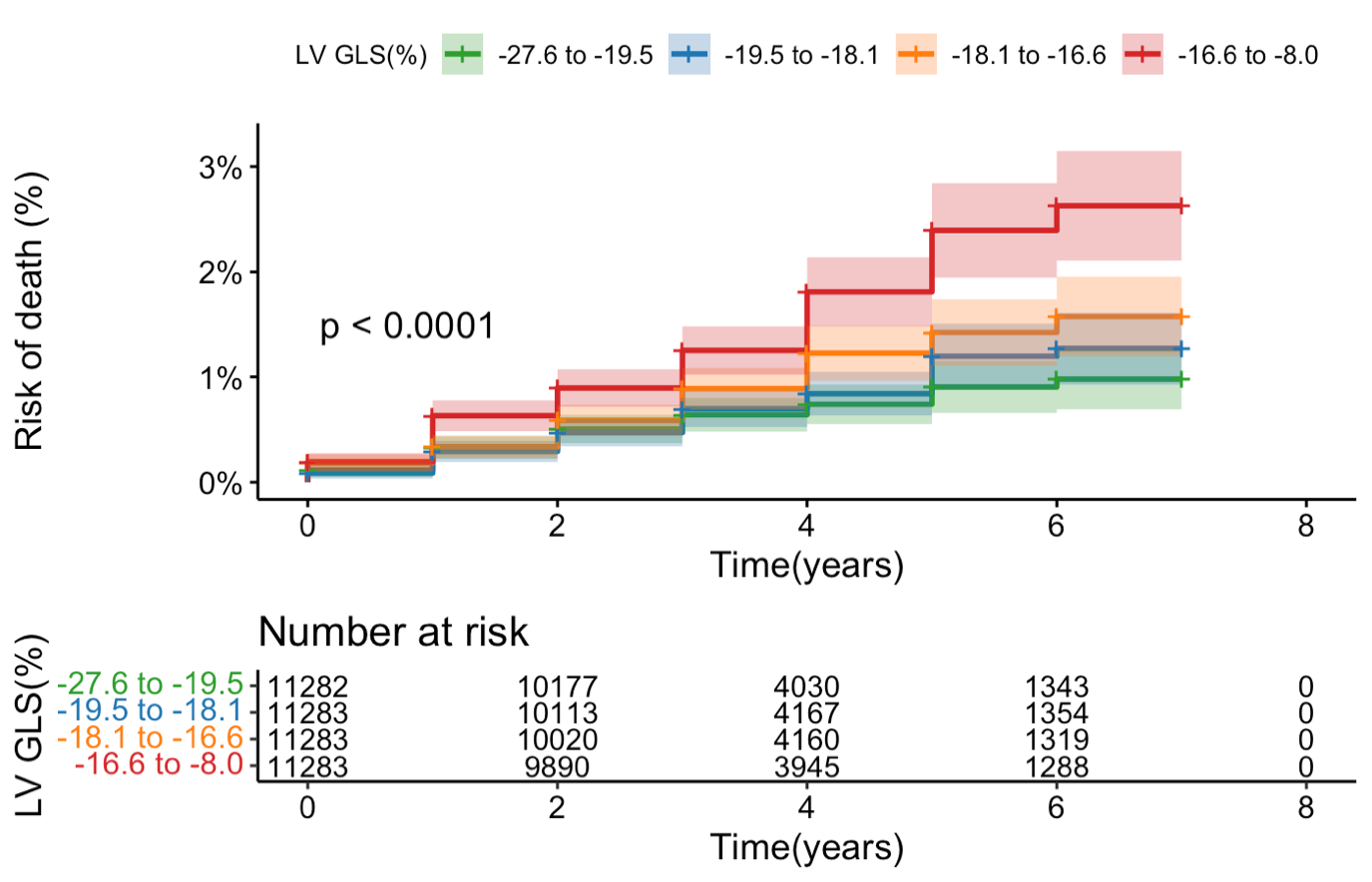


A survival plot from univariable analysis demonstrating increased risk of death associated with deteriorating left ventricular global longitudinal strain deteriorates (LV GLS), shown divided by quartiles.

Supplemental Figure 11 - Circumferential strain predicts death


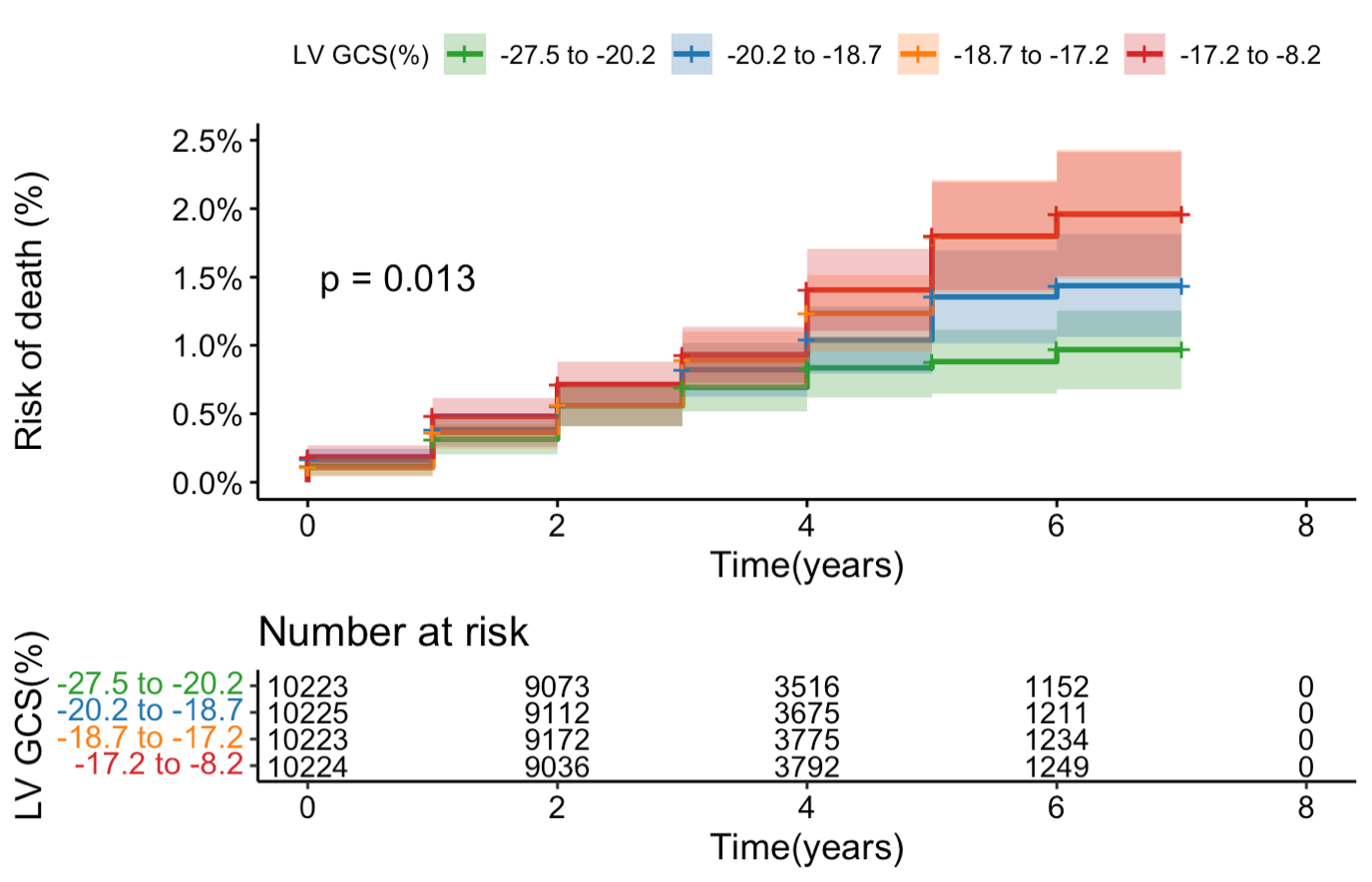


A survival plot from univariable analysis demonstrating increased risk of death associated with deteriorating left ventricular global circumferential strain deteriorates (LV GCS), shown divided by quartiles.

Supplemental Figure 12 - Radial strain predicts death


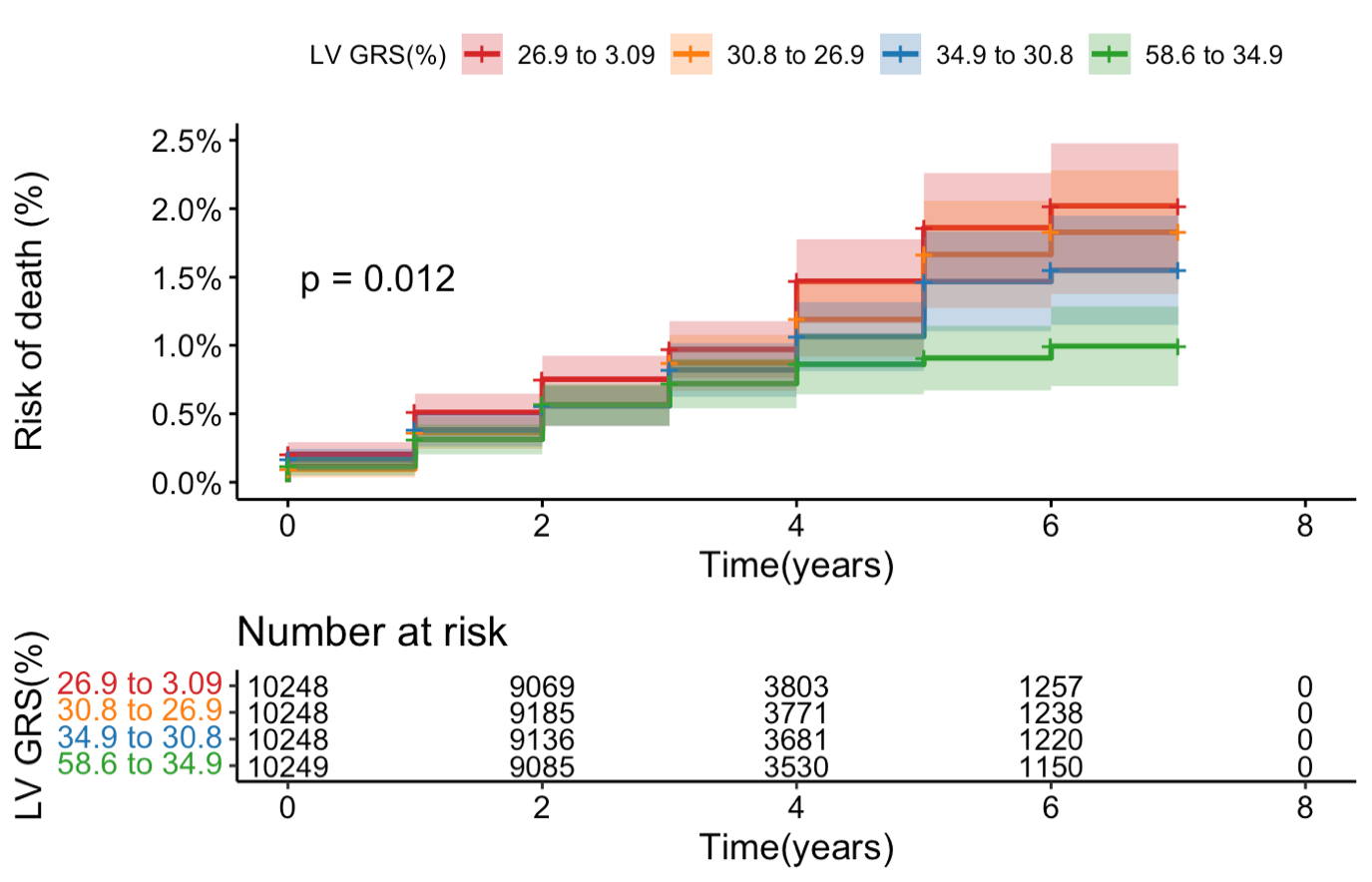


A survival plot from univariable analysis demonstrating increased risk of death associated with deteriorating left ventricular global radial strain deteriorates (LV GRS), shown divided by quartiles.
